# Using Ketamine and Propofol for Procedural Sedation of Adults in the Emergency Department: A Systematic Review and Meta-Analysis

**DOI:** 10.15171/apb.2019.002

**Published:** 2019-02-21

**Authors:** Morteza Ghojazadeh, Sarvin Sanaie, Seyed Pouya Paknezhad, Sahba-Sadat Faghih, Hassan Soleimanpour

**Affiliations:** ^1^Research Center for Evidence Based Medicine (RCEBM), Tabriz University of Medical Sciences, Tabriz, Iran.; ^2^Tuberculosis and Lung Disease Research Center, Tabriz University of Medical Sciences, Tabriz.; ^3^Emergency Medicine Research Team, Tabriz University of Medical Sciences, Tabriz, IR Iran.; ^4^Students’ Research Committee, Tabriz University of Medical Sciences, Tabriz, Iran.; ^5^Aging Research Institute, Tabriz University of Medical Sciences, Tabriz, Iran.

**Keywords:** Ketamine, Propofol, Procedural sedation

## Abstract

***Purpose:*** Ketamine-propofol combination (ketofol) is being used to provide a safe and effective procedural sedation (PS) in emergency department (ED) and may theoretically have beneficial effects since using lower doses of each drug may result in a reduction of the adverse events of both agents while maintaining optimal conditions for performing procedures. This systematic review was conducted to evaluate the efficacy, advantages and disadvantages of these two drugs for PS.

***
Methods:*** The PRISMA statement was used for this systematic review. We searched the databases of PubMed, Scopus, ProQuest, Medline (Ovid) from 1990 to August 2017 for randomized clinical trials (RCTs) in which the study population aged ≥18 and was referred to ED. Full-texts of the studies performed in adults that were published in English were reviewed for inclusion. Both authors independently evaluated all studies. Five articles were eligible for the meta-analysis based on their common outcomes.

***Results:*** The total number of subjects was 1250, of which 635 were treated with propofol and 615 were treated with ketofol. Although two of the five studies showed a better quality of sedation with ketofol, the other three did not find any significant difference between propofol and ketofol. This systematic review found a lower incidence of respiratory adverse effects in ketofol group than propofol group.

***Conclusion:***Ketamine/propofol mixture (ketofol) has less respiratory adverse effects than propofol alone in ED procedural sedation.

## Introduction

Sedation is a state that the patient’s level of consciousness reduces in order to decrease irritation, nervousness and agitation.^1^ Procedural sedation (PS) is commonly used in the emergency department (ED) to facilitate the performance of a procedure.^2,3^ The ideal drug for the PS in the ED is a medication with rapid onset of action, short duration of action, short recovery time, and minimal associated risks.^4^ Short duration of action will result in rapid recovery in the ED which is a very good aspect of the drug used for PS in the ED.^5^ The two drugs assessed in this study are propofol and ketamine. Propofol with the formulation of 2, 6-diisopropylphenol is a short-acting intravenous anesthetic drug produced in 1975.^6^ In addition, propofol was used in the ED in 1996 for PS.^7^ Propofol is a derivate of short-acting alkylphenols that is used to induce and maintain anesthesia and also to sedate the patient in the procedures. This feature has led propofol to be used for more than a decade and is widely used in the ED.^8^ The pharmacologic mechanism of propofol is related to its agonist properties on the gamma-aminobutyric acid receptor.^9,10^ The high inclination of propofol to gamma-aminobutyric acid receptors causes a remarkable pain reduction.^11^ In addition, propofol suppresses sympathetic activity and inhibits the baroreceptor reflex.^12^ It also stimulates nitric oxide production that causes vasodilation.^13^ Ketamine, a phencyclidine derivative, was first synthesized in 1962 and is commonly used for PS and analgesia and also provides excellent anesthetic induction, and maintenance.^14^ Ketamine causes dissociation between the cortical and limbic systems and thus prevents patients from perceiving sensory stimuli. It is an amnestic and analgesic agent, maintains pharyngeal reflexes, and stimulates cardiovascular tone which leads to increased blood pressure and myocardial oxygen demand.^15^ Ketamine affects several receptors including *N*-methyl-D-aspartate receptors, opioid receptors, and monoaminergic receptors. In high concentrations of ketamine, muscarinic receptors are blocked and neurotransmission through gamma-aminobutyric acid receptors is facilitated.^14^ Ketamine is used intravenously, intramuscularly, intradermally, orally, nasal and rectal. It is also used epidural or intrathecal diluted in a preservative free solution. Ketamine can be considered as adjunct/supplement to regional or local anesthesia, thereby enhancing the effectiveness of regional anesthesia. In addition, it is increasingly used for short term painful procedures in the ED with doses of 0.1-0.6 mg/kg.^14^ Currently, ketamine and propofol are used in the ED as a sedative drug in short procedures. Our goal in this study was to evaluate the efficacy and the benefits and disadvantages of these two drugs based on a systematic review.

## Materials and Methods

### Study protocol

A systematic review of databases was conducted to find randomized clinical trials (RCTs). The PRISMA statement was used for this systematic review. The search for databases, the selection of studies, the quality of studies, and the extraction of data were done by two researchers. In cases of discrepancy between the two researchers, the subject was discussed and consulted with a third reviewer.

### Inclusion and exclusion criteria

Inclusion criteria for the studies were as following: 1. RCTs comparing the efficacy of ketamine and propofol in pain relief; 2. Articles in which the study population aged ≥18 and was referred to the ED; 3. The published articles from 1990 to August 2017; 4- Articles in English language. Exclusion criteria were as following: 1. Articles in any language other than English; 2. Articles that did not have enough quality; 3. Articles conducted in animals; 4. Qualitative articles; 5. Articles with incomplete information; 6. Review articles, case reports and letters to the editor; 7. Articles published before 1990; 8. The articles with the study population of under the age of 18.

### Information databases and search strategy

We searched the databases of PubMed, Scopus, ProQuest, and Medline (Ovid). Keywords were selected based on Mesh terms using OR and AND operators and included patients, emergency medicine, emergency department, emergency service, ketamine, 2- (2-Chlorophenyl) -2- (methylamino) cyclohexanone, CI-581, CI 581, CI581, Ketalar, Ketaset, Ketanest, Calipsol, Kalipsol, Calypsol, Propofol, Disoprofol, 2,6-Bis (1-methylethyl) phenol, Diisopropylphenol, -2.6 Diprivan, Disoprivan, Fresofol, ICI-35,868, ICI 35,868, ICI 35,868, ICI-35868, ICI 35868, ICI35868, Ivofol, Recofol, Aquafol, pain score, conscious sedation, procedural sedation, moderate sedation, analgesia, and minimal sedation. Related references in the selected studies were searched manually. Gray literature and studies presented at conferences were also searched. Experts in the topic were contacted to get information about published and non-published studies.

### Selection of studies and data extraction

The articles extracted from the databases using the mentioned keywords were selected in 3 stages by the subject specialist. At first, the titles of all articles were reviewed and articles that were not consistent with the study objectives were excluded. The abstract and the full text of the articles were studied and the studies with exclusion criteria and poor association with the study objectives were identified and abandoned. Selected studies were assessed for bias risk by two evaluators using the Cochrane checklist and the discrepancies between the two evaluators were referred to the third person and eventually entered the RevMan software version 5.3.

The information extracted from the articles was summarized in the data extraction form including: first author, year of publication, country of study, type of interventions, number of people in the control and intervention group, type of study, performed procedure, intervention effectiveness and side effects of interventions. The Endnote X5 resources management software was used to organize, study the titles and abstracts, as well as to identify duplicate case.

### Statistical analysis

The number of people examined in each group and the number of people with the outcome in each group were extracted from the articles. For each study, risk ratio was calculated. A meta-analysis was used to combine the results. The relative risk of the outcome was obtained in propofol and propofol-ketamine groups. The heterogeneity between studies was investigated by Cochrane (Q) and I^2^ statistics, which expressed the percentage of variations between studies. I^2^ values less than 25% indicates low heterogeneity, between 25% and 75% shows average heterogeneity and over 75% indicates high heterogeneity. In the case of heterogeneity, the random effects model was used to calculate the overall effect size. The funnel plot and Egger regression tests were used to assess the publication bias. Statistical analysis was performed using CMA v.2.0 software and *P* value less than 0.05 was considered as a significant level.

## Results

### Articles characteristics

In the systematic search of databases, 916 titles were found that 305 titles were selected in the original review by two individuals. As we wanted to select the RCTs, 185 items which were not RCTs, were excluded. Since the title of the study was the efficacy of ketamine and propofol in adults, articles related to children were deleted (61 items). Twenty-six articles were excluded due to inaccessibility to their full texts. Extraction table was arranged based on the outcomes of each study and common outcomes were ultimately collected. Twenty-eight studies were excluded from the table as they did not have a common outcome. At the end, 5 articles were included.

**Figure 1:**
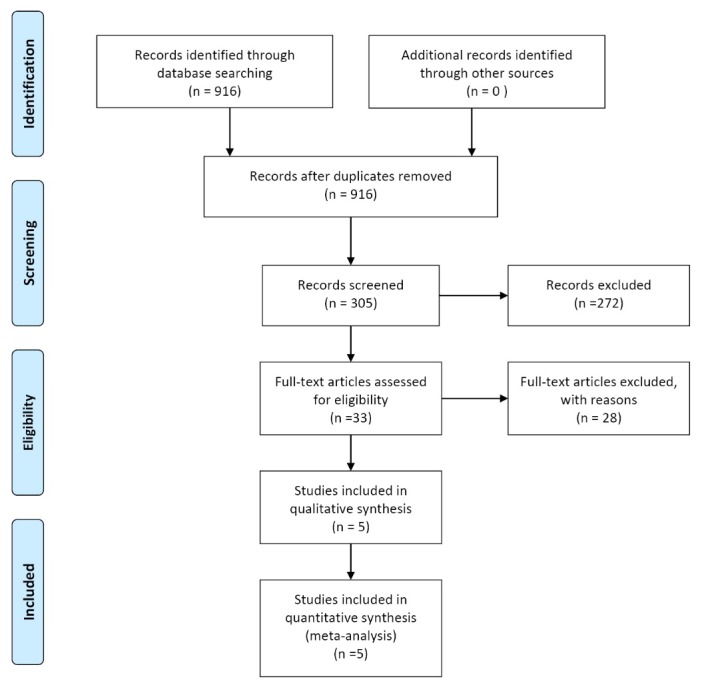
Flow diagram of the systematic literature search‏.

The graph of the articles was identified and entered into the study which is shown in Figure 1. The characteristics of the studies are shown in Tables 1 and 2.

**Table 1:** Characteristics of the study

**Author**	**Year**	**Country**	**Study Type**	**Type Blinding**	**Sample Size**		**Group A**	**Group B**	**Initial dose of Propofol**	**Initial dose of Ketofol**
					**Group A**	**Group B**				
Ferguson et al (2016)	2016	Australia	Randomized-double-blind clinical trial	2	292	281	Propofol	Ketamine + propofol	1.3 mg/kg	1.3 mg/kg ketamine and propofol combined
Miner et al (2015)	2015	USA	Randomized-double-blinded trial	2	90	85	Propofol	Ketamine + propofol	1 mg/kg	1:1—0.5 mg/kg ketamine and propofol combined; 4:1—0.8 mg/kg ketamine, 0.2 mg/kg propofol
Andolfatto et al (2012)	2012	Canada	Randomized- double-blind trial	2	142	142	Propofol	Ketamine + propofol	0.75 mg/kg	0.375 mg/kg ketamine and propofol combined
David et al (2010)	2010	Columbia	Randomized- double-blind- placebo-controlled trial	2	97	96	Propofol	Ketamine + propofol	1 mg/kg	0.5 mg/kg ketamine and propofol combined
Phillips et al (2010)	2010	USA	Prospective- randomized single blind	1	14	11	Propofol	Ketamine + propofol	0.5–1.5 mg/kg	0.75 mg/kg ketamine and propofol combined

**Table 2:** Patients distribution in reviewed studies

**Author**	**Procedure**								**Intervention**				**Outcome**			
	**Orthopedic procedures**		**Incision and drainage of abscess**		**Cardioversion**		**Chest tube placement**		**Airway repositioned/opened**		**Assisted ventilation (bag-valve-mask)**		**Desaturation**		**Apnea (loss of ETCO2 15 s)**	
	**Group A**	**Group B**	**Group A**	**Group B**	**Group A**	**Group B**	**Group A**	**Group B**	**Group A**	**Group B**	**Group A**	**Group B**	**Group A**	**Group B**	**Group A**	**Group B**
Ferguson et al (2016)	176	175	59	57	25	27			34	27	9	3	23	17	16	11
Miner et al (2015)	36	81	52	94	1	1	1	5	13	5	8	3			11	6
Andolfatto et al (2012)	86	85	23	28	21	17	6	3	14	5			36	38		
David et al (2010)	85	84	3	2			0	1	9	8	5	2	11	7	4	2
Phillips et al (2010)	14	11											0	0	0	0

Abbreviation: ETCO2, end-tidal carbon dioxide.

### Quality evaluation of the articles

**Figure 2:**
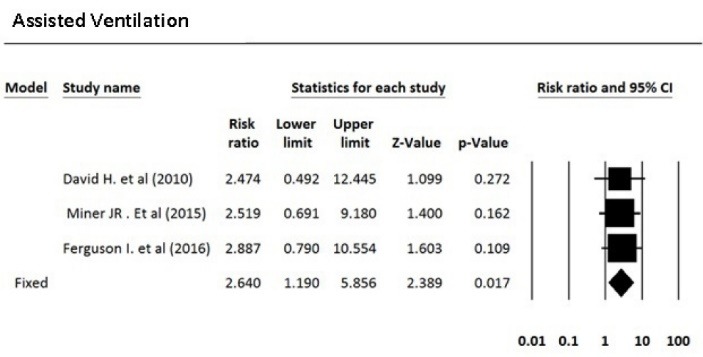
Need for assisted ventilation in patients sedated with ketofol vs. propofol.

**Figure 3:**
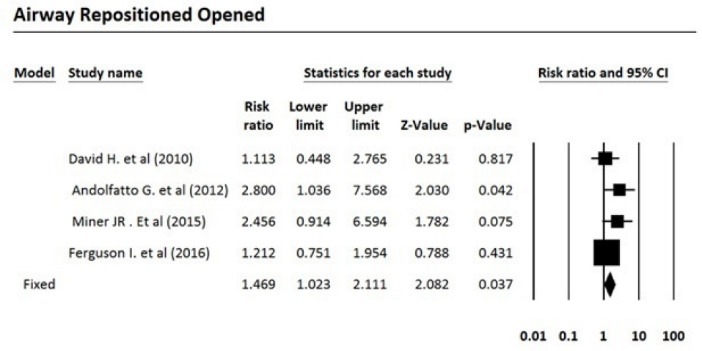
Need for airway repositioning maneuvers in patients sedated with ketofol vs. propofol.

**Figure 4:**
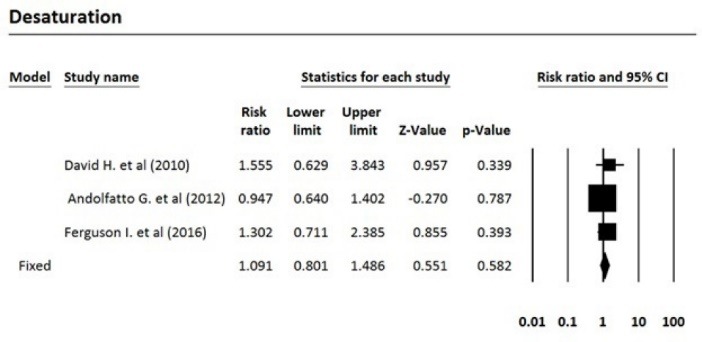
Risk of desaturation in patients sedated with ketofol vs. propofol.

**Figure 5:**
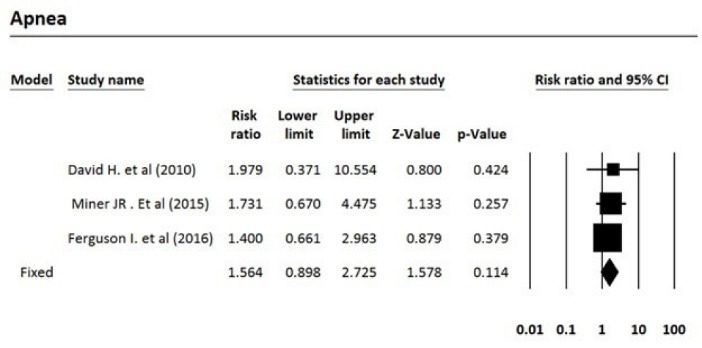
Risk of apnea in patients sedated with ketofol vs. propofol.

Criteria for making judgments about assessing the risk of bias in the Cochrane checklist include random sequence generation, allocation concealment, blinding of participants and personnel, blinding of outcome assessment, incomplete outcome data, selective reporting, and any other bias. Figures 2- 5 show the results of the evaluation of the quality of articles entered into this meta-analysis using the Cochrane tool. The word “yes” means a low risk of bias, the word “no” means a high risk of bias and the term “unclear” means that there is not enough information to judge the risk of bias.

### Meta-analysis

After reviewing the selected articles, five articles were eligible for meta-analysis. The total number of subjects was 1250, of which 635 were treated with propofol and 615 were treated with propofol plus ketamine. Based on the fixed effects model, the pooled risk ratio for comparing the incidence of airway repositioned/opened, apnea, assisted ventilation, and desaturation for the propofol-treated group was 1.47, 1.57, 2.64, and 1.09 times more than the group treated with propofol plus ketamine, respectively. Table 3 indicates the meta-analysis results with 95% confidence interval for risk ratio and the level of heterogeneity of the studies entered into this meta-analysis. Five studies that fulfilled eligibility criteria were included in our review. These five studies were published between 2010 and 2016. Three studies had been performed in the United States, one in Canada and one in Australia. Andolfatto et al compared a 1:1 mixture of Ketamine and propofol (ketofol) with propofol alone for PS in emergency room. They studied 284 adult patients in two groups. Thirty percent of ketofol group experienced an adverse effect of drug versus 32% of propofol group. They found no significant different in adverse effects, need for medication re-administration and need for bag-mask ventilation. No significant difference has been found in induction time or duration of sedation. However, depth of sedation was more consistent with ketofol. The primary outcome in this study the respiratory adverse event as described by the Quebec criteria; however, the limitation of this criteria is that the decision for intervention is dependent to the judgment of the clinician. Moreover, adverse events may differ based on the ratio of the used ketamine and propofol.^16^ In a randomized, double-blind, placebo controlled trial, David and Shipp studied 193 ED patients in two groups. Both children and adult patients received 1 mg/kg propofol in one group. In the other group, 0.5 mg/kg of ketamine was added to the regimen. They found same incidence of respiratory adverse effects in two groups (22% for ketofol vs 28% for propofol) but a better satisfaction and sedation quality in ketofol group. Although maintenance of the blinding of the study was a challenge and a limitation for the study because of ketamine’s side effects such as nystagmus, authors did not find it a significant confounding factor in their results.^17^ Philips et al studied 28 adult trauma patients who needed deep sedation for bone fracture manipulation in a level one trauma center ED. Fourteen subjects who received 0.5 to 1.5 mg/kg propofol were compared with 14 subjects who received 0.75 mg/kg propofol and 0.75 mg/kg ketamine. Propofol group had a lower systolic blood pressure (SBP) during sedation and had a statistically greater decrease in SBP. Ketofol group had a higher bispectral index. No significant respiratory adverse effect was found in the groups. A slightly higher degree of pain and recall was reported in propofol group. Small sample size was an important limitation in this study.^18^ Ferguson et al studied 573 adult participants in two groups. In a double blind randomize clinical trial, 292 emergency patients received propofol for sedation versus 281 patients receiving 1:1 mixture of Ketamine/propofol. Vital signs, depth of sedation, wake up agitation and patient satisfaction were studied during and after procedure. Both groups showed a similar incidence of respiratory adverse effects, but propofol resulted in a slightly higher rate of hypotension with doubtful clinical relevance. Both groups had a high level of patient satisfaction.^19^ Miner et al performed a double-blind RCT comparing propofol and 1:1 and 1:4 ketamine/propofol mixture; 271 subjects in three groups completed the study. There was no significant difference in respiratory adverse events between three groups and ketofol showed no benefit over propofol, neither in 1:1 nor in 1:4 mixture, in efficacy of sedation time or patient satisfaction.^20^

**Table 3:** Meta-analysis results

**Outcome/intervention**	**Effect size and 95% CI**						**Heterogeneity**			
	**Number studies**	**Risk ratio**	**Lower limit**	**Upper limit**	**Z-value**	***P*** **-value**	**Q-value**	***df*** **(Q)**	***P*** ** value**	**I** ^ 2 ^
Airway repositioned/ opened	4	1.47	1.02	2.11	2.08	0.037	3.64	3	0.30	17.48
Apnea	3	1.56	0.90	2.72	1.58	0.114	0.20	2	0.90	0.00
Assisted ventilation	3	2.64	1.19	5.86	2.39	0.017	0.03	2	0.99	0.00
Desaturation	3	1.09	0.80	1.49	0.55	0.582	1.42	2	0.49	0.00

## Discussion

Both propofol and ketamine are used for PS worldwide.^21^ Propofol has a lower recovery agitation incidence and shorter half-life but respiratory side effects like hypoxia and respiratory depression limit its use. In the other hand, ketamine has an advantage of respiratory function preservation and lower respiratory side effects.^16^ Thus, theoretically combination of these two drugs can reduce their disadvantages and provide a better result.

Although two of five studies showed a better quality of sedation with ketofol^17,18^ others did not find a significant difference between propofol and ketofol.^16,19,20^ This systematic review found a lower incidence of respiratory adverse effects in ketofol group than propofol group. We could not find a significant difference in hemodynamic profile of two drugs. Although Philips et al showed a lower SBP and larger SBP reduction in propofol group versus ketofol but other studies with larger sample size did not find same results.^16-18,20^ Some of the side effects of propofol include hypotension, asystole, bradycardia, and dose-dependent respiratory depression. Its low side effects has made it as a selective drug in many medical procedures that do not require general anesthesia.^22^ Propofol is contraindicated in patients who have allergies to propofol, egg, or soy protein.^10,22^ Some side effects of ketamine include hypertension, tachycardia, and liver and kidney toxicity in overdose of ketamine. Ketamine is contraindicated in patients with ophthalmologic disorders, ischemic heart disease, vascular aneurysm, schizophrenia, and a history of hypersensitivity to ketamine.^14^ Ketamine is frequently used in the ED for sedation in procedures and intubation.^23^ The opposite physiologic outcomes of ketamine and propofol are the option for synergy, and this has been a reason for their combined use, as “ketofol,” to facilitate PS in the ED.^16^ The evidences show that Ketofol is effective for PS in the ED, and it may have less adverse effects than sole propofol.^19^ It is shown that Ketofol can decrease respiratory depression, vomiting, and recovery duration because of the counterbalance effects of the drugs on each other.^20^

## Conclusion

We concluded that ketamine/ propofol mixture (ketofol) has less respiratory adverse effects than propofol alone in ED procedural sedation. Further research is needed in this field to determine the efficacy of this combination for PS.

## Ethical Issues

Not applicable.

## Conflict of Interest

The authors declare no conflict of interest.

## Acknowledgments

The authors are grateful to all the health staff who participated in the study, in addition to the staff of the RDCC, Tabriz University of Medical Sciences, Iran. This article is based on a dataset forming part of Sahba-Sadat Faghih’s M.D thesis, entitled “Ketamine and Propofol sedation in the adults and use of it by emergency medicine specialists: A systematic review and meta-analysis”. It is registered at Tabriz University of Medical Sciences (No: 57898) in 2016.
